# Application of the moving epidemic method for influenza surveillance in Kunming

**DOI:** 10.3389/fpubh.2025.1601781

**Published:** 2025-06-25

**Authors:** Yudong Gao, Yang Zhou, Siyi Luo, Zhengji Chen, Yujue Wang, Zhiyan Zha, Xiaoxiao Song

**Affiliations:** ^1^Kunming Center for Disease Control and Prevention, Kunming, Yunnan Province, China; ^2^School of Public Health, Kunming Medical University, Kunming, Yunnan Province, China

**Keywords:** moving epidemic method, MEM, influenza surveillance, epidemic thresholds, epidemic intensity

## Abstract

**Objective:**

The Moving Epidemic Method (MEM) has been widely used to assess seasonal influenza epidemics in temperate and subtropical regions. This is the first study to validate the use of MEM in a subtropical plateau environment.

**Methods:**

This study applied the Moving Epidemic Method (MEM) to establish influenza epidemic thresholds in Kunming, China, using virological surveillance data from 2011–2012 to 2023–2024.

**Results:**

The MEM model demonstrated high sensitivity (93%) and specificity (67%), with no detection lag for the 2023–2024 season. Epidemic thresholds (8%), which were notably lower than those in other subtropical regions, may potentially be attributed to Kunming's plateau monsoon climate.

**Conclusion:**

This study underscored MEM's adaptability in subtropical plateau settings and provided actionable thresholds for early outbreak response.

## 1 Introduction

Influenza A and B are major causes of seasonal epidemics (1), contributing to a significant global health burden (2). Every year, influenza leads to 3–5 million cases and ~400,000 deaths globally (3) in addition to over 5 million hospitalizations. The disease burden of influenza is particularly severe during epidemic seasons, especially among vulnerable populations, such as older adults (4), young children (5), and individuals with underlying health conditions (6). In China (7), the morbidity rate of influenza in 2023 was 906.56 cases per 100,000 people, making it the most prevalent of the 41 diseases reported nationally. The estimated mortality rates due to influenza were 14.33 per 100,000 across all age groups and 122.79 per 100,000 among those aged 65 and older (8).

Four primary strains of the influenza virus—A(H1N1)pdm09, A(H3N2), B/Victoria, and B/Yamagata—have been circulating in human populations for decades (9). However, B/Yamagata has been rarely detected since March 2020 (10). Meanwhile, genetic diversity in H1N1pdm09 and B/Victoria has been increasing since April 2021, whereas H3N2 diversity has declined more gradually (10). Despite these genetic variations, the seasonal patterns of each influenza virus remain insufficiently explored (11, 12). In temperate regions, influenza typically peaks in winter, whereas subtropical and tropical regions exhibit less pronounced seasonality (13, 14). For example, northern China experiences a single seasonal influenza peak, whereas southern China often sees both a major and a minor peak (15). These regional variations are influenced by the distribution and characteristics of circulating viruses (16), making predicting epidemic timing and intensity challenging (17).

Therefore, determining the onset of an influenza epidemic is crucial for implementing effective public health interventions. Since 2004, China has implemented the China Infectious Disease Automated-Alert and Response System (CIDARS) to monitor influenza epidemics (18). Although climate change and mutations introduce uncertainties, seasonal influenza still exhibits repeatability and structural characteristics, and key patterns can be identified based on historical data. Various approaches (18, 19) have been proposed to detect influenza epidemics, including the cumulative sum (CUSUM), serial regression, and Moving Epidemic Method (MEM), among others. The MEM initially proposed by Vega et al. (20) has been widely recognized as an effective tool for defining epidemic and non-epidemic periods based on routine surveillance data. MEM has been well-documented in temperate (21–23) and subtropical regions (24, 25). Similarly, the results showed that the prediction error from the actual epidemic week was only ±1 week in Respiratory Syncytial Virus (RSV) (26) verifying the robustness of MEM in different diseases and data sources.

To date, the potential application of MEM in detecting influenza epidemics in subtropical plateau regions remains underexplored. This study is the first to validate MEM in a subtropical plateau city.

## 2 Materials and methods

### 2.1 Study area and data sources

**Figure 1:**
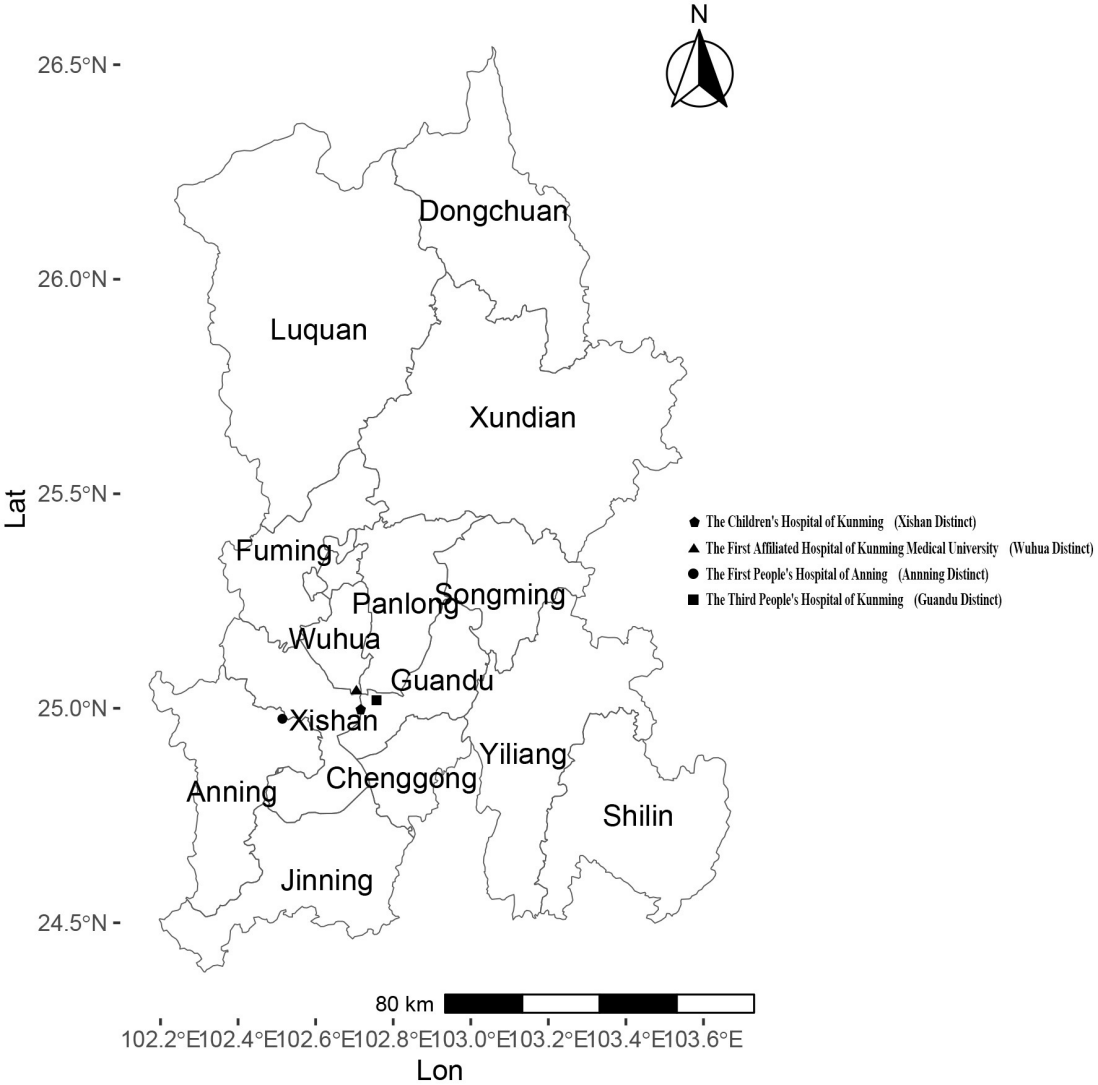
Distribution of 14 counties/districts and 4 sentinel hospitals in Kunming.

Kunming, located in Southwestern China, is the capital city of Yunnan Province with a resident population of 8.69 million as of the end of 2024. Administratively, Kunming consists of 14 county-level divisions, including 7 districts, 6 counties, and 1 county-level city. The four sentinel surveillance sites were established in response to the 2003 following Severe Acute Respiratory Syndrome (SARS) outbreak, based on two considerations: (1) multiple administrative levels of hospitals, including provincial, municipal, and county-level facilities, and (2) key hospital types: general hospitals, children's hospitals, and infectious disease specialty hospitals. By the end of 2024, four national sentinel hospitals remained unchanged from their establishment. They were actively conducting influenza surveillance: the First Affiliated Hospital of Kunming Medical University (102key ho 25 county the Third People's Hospital of Kunming(102he Thi 25nming(1 the Children's Hospital of Kunming(102en's H 24nming(1 and the First People's Hospital of Anning (102 the F 2402 the (Figure 1). Each sentinel hospital collected a minimum of 20 respiratory specimens per week from individuals presenting with influenza-like illness (ILI). An influenza-like illness (ILI) case was defined as a fever (body temperature ≥ 38°C) accompanied by either a cough or a sore throat. The collected specimens were sent to reference laboratories for influenza virus analysis, where reverse transcription polymerase chain reaction (RT-PCR) was conducted to determine influenza virus subtypes within the same week of sample collection. The laboratory results were electronically submitted to the web-based National Influenza Surveillance Information System (NISIS). The confirmed influenza case data, sourced from China's infectious disease surveillance system (CIDS), and demographic data come from the official website of the National Bureau of Statistics.

Based on historical seasonal epidemic patterns in Kunming, influenza is most prevalent during the winter and spring months. To optimize the MEM analysis by focusing on epidemiologically meaningful data, we defined the monitoring period from the 40th week of the current year to the 30th week of the following year, covering a total of 43 weeks per influenza season. This period encompasses the entire phase of substantial influenza transmission in Kunming, excluding weeks 31–39, during which there was minimal activity. In addition to the overall influenza positivity proportion (PR), the weekly PRs of A(H1N1)pdm09, A(H3N2), B/Victoria lineage, and B/Yamagata lineage were also calculated separately. This study included 10 influenza seasons from 2011–2012 to 2019–2020, as well as the 2023–2024 season, for analysis using the MEM.

### 2.2 Cross-correlation analysis

A spatiotemporal analysis was conducted to examine the geographic and temporal distribution of influenza incidence in Kunming from 2010 to 2020. To assess the temporal relationship between PR and incidence rate, a cross-correlation function (CCF) analysis was conducted using weekly data from 2010 to 2024. Pearson correlation coefficients were calculated at lags from −5 to +5 weeks to determine whether PR could serve as a leading indicator of incidence rate. The analysis was performed across four periods: the full dataset (2010–2024), the pre-pandemic years (2010–2019), the post-pandemic years (2023–2024), and a filtered set excluding 2020–2022.

### 2.3 Moving epidemic method

The Moving Epidemic Method (MEM) is a three-step process used to define the start, length, and end of each influenza epidemic season.

**Step 1: Determining the Epidemic Period**.

The epidemic period is defined as the time when the cumulative monitoring index first drops below a predefined threshold δ, expressed as a percentage of the total cumulative monitoring index for the season. The length of the epidemic period is determined using the Maximum Accumulated Percentage (MAP), which identifies the minimum number of consecutive weeks that account for the highest cumulative percentage of influenza cases, as described by Vega et al. (20), each influenza season is divided into three distinct periods: the pre-epidemic period, the epidemic period, and the post-epidemic period. In this study, MAP represents the maximum value of the cumulative percentage of PR over a specific period within a flu season, relative to the total cumulative positivity rate for the entire season. The particular calculation formulas are as follows:


(1) $$t_{j}^{r}=\underset{k=1,\ldots ,S-r+1}{\max}\left\{\sum_{i=k}^{k+r-1}t_{i,j}\right\},\forall_{r}=1,,S$$

(2) $$\begin{array}{l} t_{j}^{s}=\overset{S}{\underset{i=k}{\sum }}t_{i,j} \end{array}$$

(3) $$\begin{array}{l} P_{j}^{r}=\frac{t_{j}^{r}}{t_{j}^{s}} \end{array}$$

The MAP curve draws the maximum cumulative rate for a period of a given length r, expressed as a percentage of the total rate of ${\text{P}}_{\text{j}}^{\text{r}}$ of the total rate of the j season. The ${\text{t}}_{\text{j}}^{\text{r}}$ is the highest accumulated rate among j epidemic in r period. The ${\text{t}}_{\text{j}}^{\text{s}}$ is the accumulate rate in the j season. The **t**_**i**, **j**_ is the *i* rate of the *j* season, and *S* is the monitoring weeks in each season. The *k* is the number of start weeks of consecutive r weeks.

**Step 2: Calculation of Epidemic Thresholds**

The epidemic thresholds are calculated based on PR from both the pre-epidemic and post-epidemic periods. The top *n-*values (where *n* = 30/N) with the highest PR in both the pre-epidemic and post-epidemic periods are selected for analysis. The 95% one-sided confidence interval (CI) upper limit of the arithmetic mean of these 30 values is used to define the Pre-epidemic and post-epidemic thresholds.

**Step 3: Classification of Epidemic Intensity**

The epidemic intensity is categorized based on PR values observed during the epidemic period. The top *n-*values (where *n* = 30/N) with the highest positivity rates in each epidemic period are selected for analysis. The 40, 90, and 95% one-sided confidence intervals (CIs) upper limits for the geometric mean of these 30 values are used to define the thresholds for: moderate intensity, high intensity, and very high intensity.

### 2.4 Model evaluation

The MEM model can be used to classify each season into three periods, namely, pre-epidemic, epidemic, and post-epidemic periods. The rates in the pre-epidemic period were expected to be lower than the pre-epidemic threshold rate, whereas rates during the epidemic period were expected to be higher. Rates in the post-epidemic period were expected to be lower than the post-epidemic threshold rate. For the 2023–2024 season, the epidemic threshold and intensity levels (moderate, high, and very high) were determined based on historical pre-epidemic, post-epidemic, and epidemic values from the 2011–2012 to 2019–2020 seasons.

The MEM model was implemented using RStudio 4.2.1, and its performance was assessed through calculations of sensitivity, specificity, and timeliness. A true positive week is defined as a week within the epidemic period when the PR exceeds both the start- and post-threshold. A true negative week is a week within the pre- or post-epidemic period where the PR is below the threshold. Sensitivity is calculated as the number of true positive weeks divided by the total number of weeks in the epidemic period. Specificity is the number of true negative weeks divided by the total number of weeks in the non-epidemic period. Youden's index is the sum of sensitivity and specificity minus one. Timeliness was defined as the number of weeks between the first occurrence of a positive proportion exceeding the established epidemic threshold and the first week of the MEM-defined epidemic period. The threshold parameter δ was iteratively adjusted, with an initial value of 1.0, a final value of 3.0, and a step size of 0.1. The optimal value of δ was selected based on its performance in terms of sensitivity, specificity, and Youden's Index.

The optimized MEM model was subsequently employed to determine the epidemic threshold and intensity thresholds, which were then applied to assess the epidemic intensity of the 2023–2024 winter-spring influenza season in Kunming. For a target type of influenza virus, an epidemic threshold was calculated using the seasons when the proportion of the target strain exceeded 25%.

### 2.5 Cross-validation analysis

The cross-validation procedure was employed to evaluate the performance of MEM. Each influenza season is treated individually as the target season, while other seasons serve as a historical baseline for comparison. The MEM model's first and second steps are applied to calculate the epidemic start- and post-thresholds for the target season. The actual weekly PR during the season was compared with the pre-epidemic, epidemic, and post-epidemic periods, as well as the start and post-thresholds determined by the MEM model. Sensitivity, specificity, and Youden's index are then calculated.

## 3 Results

### 3.1 Seasonal influenza activity in Kunming

**Figure 2:**
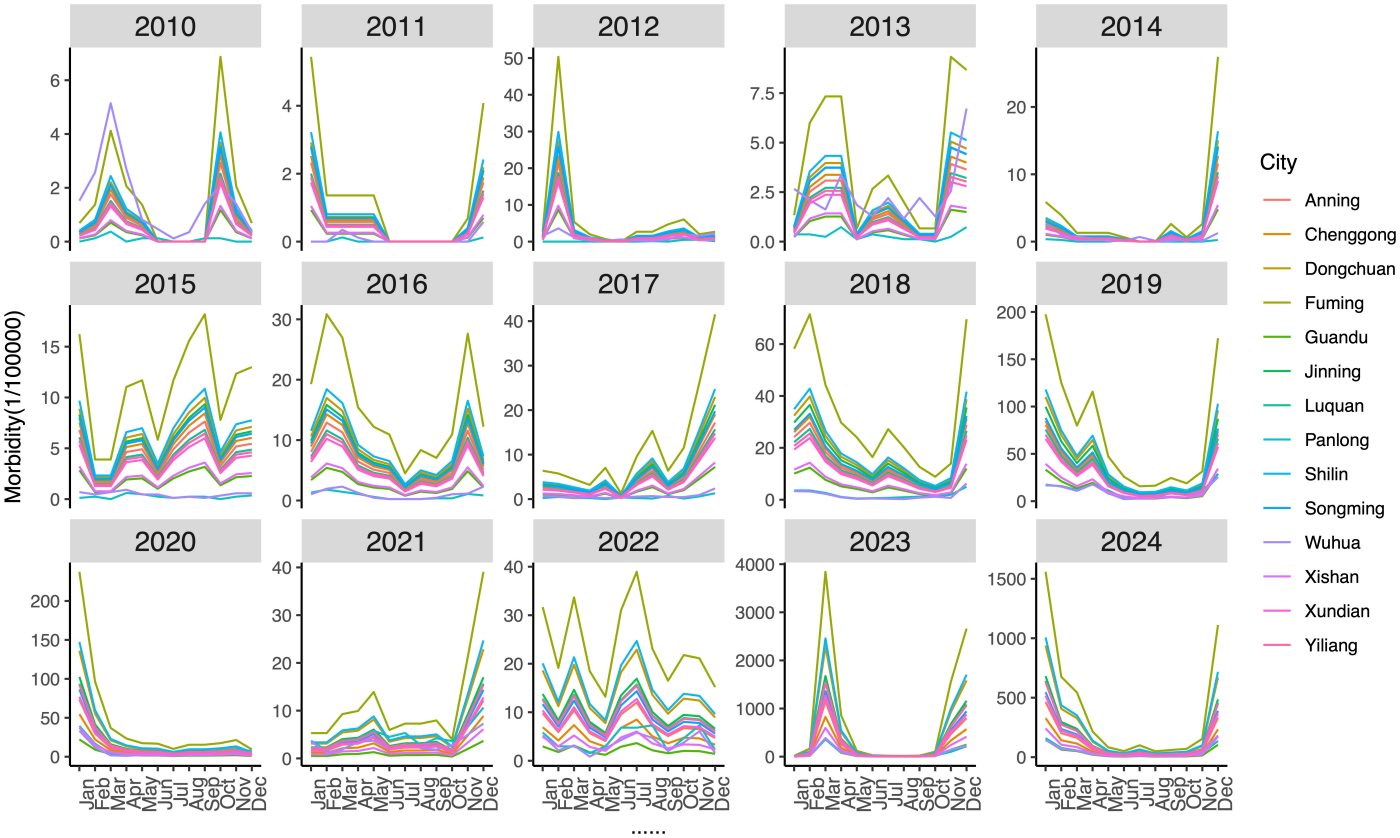
Time series of influenza incidence rate in Kunming, 2010–2024.

**Figure 3:**
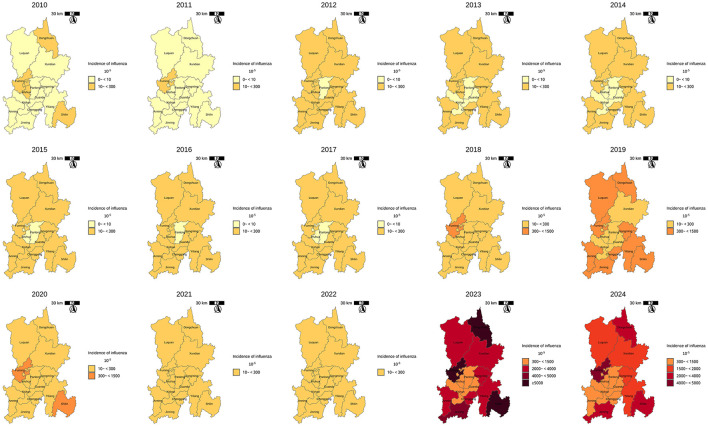
Spatial distribution of influenza incidence rate in Kunming, 2010–2024.

The annual and seasonal analysis of influenza incidence rate across 14 districts and counties of Kunming from 2010 to 2024 revealed substantial heterogeneity in both intensity and distribution over time. Time–series plot (Figure 2) shows a clear seasonal pattern, with influenza incidence typically peaking during the winter and early spring months (January to March). From 2010 to 2018, morbidity levels in most regions remained relatively low and stable, with peak values generally below 500 cases per 100,000 population. However, a sharp increase in incidence was observed after 2018, particularly in peripheral districts–countries, Fuming, Shilin, Dongchuan, and Jingning. The decline in morbidity between 2020 and 2022 is notable across all districts, aligning with the period of coronavirus disease 2019 (COVID-19) control measures, which likely reduced the spread of influenza. However, this was followed by an unprecedented resurgence in 2023–2024, with many peripheral districts reporting incidence levels exceeding 3,000–9,000 per 100,000, far above pre-pandemic norms. Spatial distribution maps (Figure 3) further highlight this post-pandemic intensification and concentration. Prior to 2018, the majority of the regions exhibited modest rates (<300/100,000), with minimal spatial disparity. In contrast, by 2023, regions such as Dongchuan, Fuming, Luquan, Shilin, and Yiliang formed a clear belt of high-morbidity clusters, particularly in the northeast and southeast of Kunming. Central urban districts, such as Wuhua, Panlong, and Guandu, maintained relatively lower incidence levels throughout the study period (Table 1).

**Table 1:** Annual influenza incidence rate per 100,000 population in 14 districts of Kunming, 2010–2025.

**City**	**2010**	**2011**	**2012**	**2013**	**2014**	**2015**	**2016**	**2017**	**2018**	**2019**	**2020**	**2021**	**2022**	**2023**	**2024**
Fuming	19.24	15.65	81.88	50.00	47.71	136.36	186.26	138.66	396.58	870.44	518.37	136.42	283.83	9,405.64	4,672.57
Shilin	11.37	9.27	48.61	29.53	28.52	81.40	111.37	82.60	237.59	520.30	321.81	86.26	179.84	6,018.03	3,018.65
Dongchuan	10.30	8.39	44.36	27.08	26.16	74.73	102.55	76.68	220.77	485.61	297.23	79.81	166.86	5,595.71	2,813.51
Jinning	9.87	8.07	41.92	25.51	24.58	70.00	95.61	70.82	202.78	439.78	223.82	59.33	123.28	4,100.86	2,043.33
Luquan	7.06	5.76	30.35	18.52	17.89	51.09	70.07	52.39	151.27	333.74	204.55	54.76	114.54	3,834.62	1,925.11
Yiliang	6.68	5.44	28.64	17.48	16.90	48.39	66.38	49.34	141.57	309.76	201.36	53.81	112.57	3,765.22	1,888.86
Songming	9.75	7.96	41.92	25.42	24.25	67.52	91.11	65.98	183.82	387.68	188.60	50.12	104.47	3,328.62	1,638.14
Xundian	6.13	5.00	26.35	16.13	15.63	44.87	61.69	45.96	131.89	289.78	168.21	44.59	92.91	3,095.99	1,545.02
Anning	8.20	6.69	34.76	21.01	20.17	57.22	77.89	57.47	164.57	355.78	160.21	42.39	87.51	2,829.29	1,392.45
Chenggong	9.01	7.26	37.89	23.01	22.12	63.25	86.23	63.69	177.57	372.95	119.32	30.97	61.90	2,022.40	982.07
Xishan	3.71	3.02	15.86	9.72	9.42	26.96	36.99	27.50	79.10	173.54	80.67	21.33	43.94	1,459.97	724.49
Wuhua	18.94	1.04	15.02	28.97	7.74	5.75	12.60	8.00	19.93	120.45	77.51	37.55	42.20	926.38	582.37
Kunming	2.52	1.06	7.15	8.28	3.67	7.65	12.69	7.99	25.43	82.19	55.11	24.06	37.45	804.86	567.40
Panlong	0.99	0.25	1.22	3.65	1.09	3.01	9.59	4.66	22.92	137.00	104.86	56.21	53.42	895.95	563.89
Guandu	3.28	2.67	14.09	8.62	8.35	23.81	32.67	24.15	68.61	146.63	48.37	12.79	26.49	880.82	438.00

**Figure 4:**
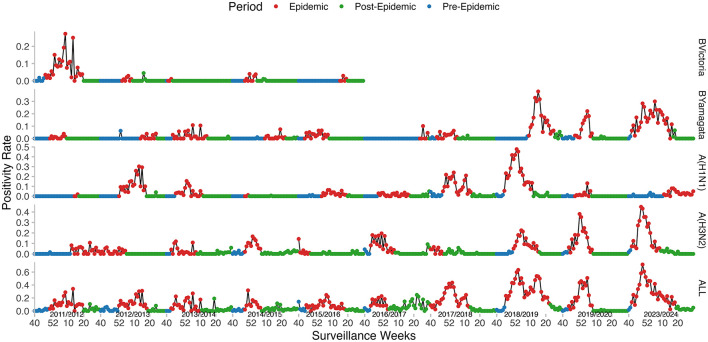
Weekly influenza positivity rates (PR) and MEM-defined epidemic periods in Kunming, from 2011–2012 to 2023–2024. Curves from bottom to top: All types, A(H3N2), A(H1N1)pdm09, B/Yamagata, and B/Victoria.

Regarding the virological data, the peak PR ranged from 24.63 to 71.62%. The weekly PR exhibited seasonal trends, typically peaking either at the beginning or the end of the year. The peak timing varied between seasons (Figure 4) and predominantly followed a unimodal distribution, except for the 2017–2018 and 2018–2019 seasons, which showed a clear bimodal pattern. The dominant influenza strains also varied from one season to another. A(H1N1)pdm09 was the predominant strain in the majority of the seasons, particularly in 2012–2013, 2013–2014, 2017–2018, 2018–2019, and 2019–2020, with positivity rates exceeding 45%. A(H3N2) was prevalent in the seasons 2014–2015, 2016–2017, 2019–2020, and 2023–2024, with a positivity rate exceeding 45% in each of these seasons. B/Victoria was the dominant strain in the 2011–2012 season, accounting for 72.6% of positive cases. B/Yamagata was most prevalent in the 2023–2024 season, making up 45.58% of positive cases (Table 2).

**Table 2:** Virological characteristics of influenza in Kunming from 2011–2012 to 2023–2024 seasons.

**Seasons**	**PR peak**		**Percentage of Positive specimens (%)**			
	**Week**	**Value (%)**	**A(H1N1)pdm09**	**A(H3N2)**	**B/Yamagata-lineage**	**B/Victoria-lineage**
2011–2012	13	34.09	0.63	20.89	4.43	74.05
2012–2013	15	31.03	81.60	12.80	5.60	0.00
2013–2014	5	27.23	45.26	29.93	24.82	0.00
2014–2015	50	31.76	1.39	86.11	12.50	0.00
2015–2016	6	24.63	35.96	20.18	34.21	9.65
2016–2017	22	24.74	16.96	75.45	7.14	0.45
2017–2018	2	43.64	69.15	14.54	11.70	4.61
2018–2019	2	63.28	54.45	19.28	25.82	0.45
2019–2020	4	50.82	8.43	66.01	25.56	0.00
2023–2024	49	71.62	9.34	45.08	45.58	0.00

### 3.2 Cross-correlation analysis

**Figure 5:**
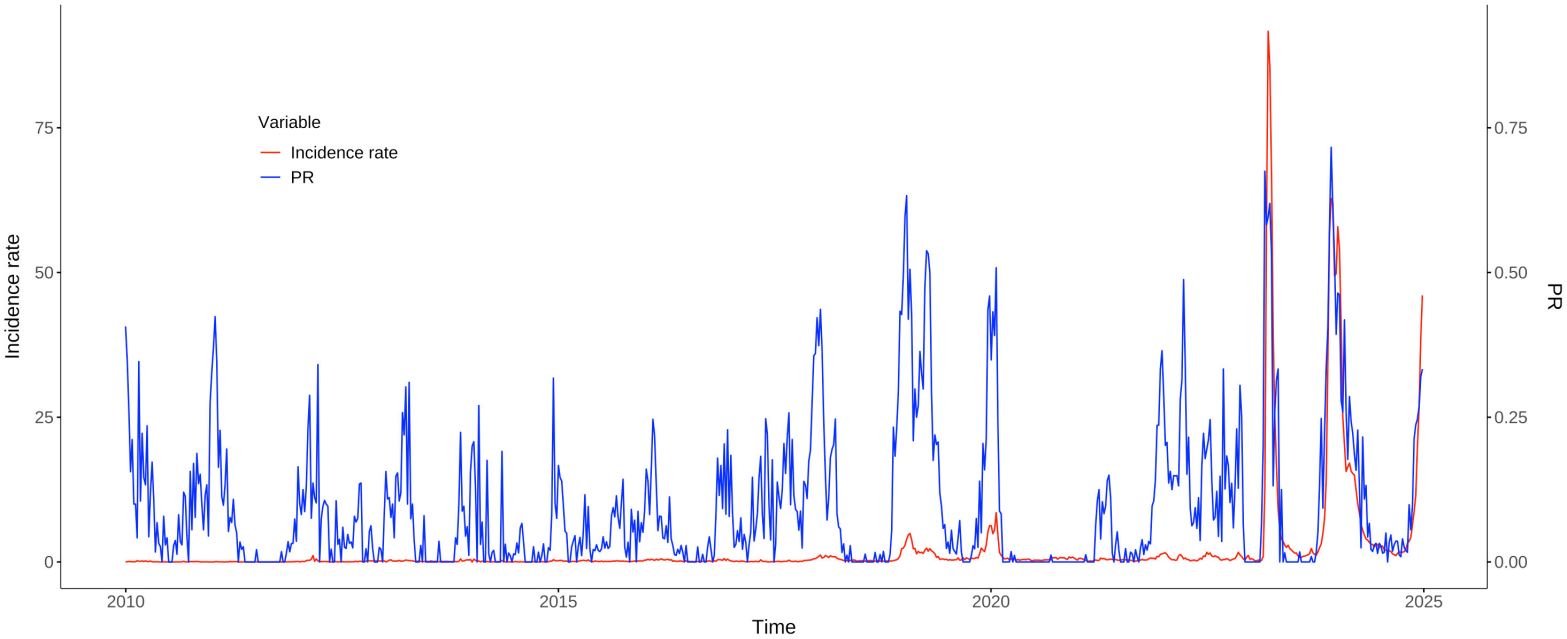
Temporal trends of influenza incidence rate and PR in Kunming, 2010–2025.

The cross-correlation analysis demonstrated that PR consistently showed a statistically significant positive correlation with incidence rate, often with a one-week lead. During the whole study period from 2010 to 2024, the strongest correlation (*r* = 0.54) was observed at a lag of −1 week. When the COVID-19 pandemic years (2020–2022) were excluded, the correlation increased slightly (*r* = 0.57), still at a lag of −1 week. In the pre-COVID-19 pandemic period (2010–2019), the peak correlation (*r* = 0.59) occurred at a zero lag, indicating a contemporaneous relationship. In contrast, the post-COVID-19 pandemic period (2023–2024) showed a robust correlation (*r* = 0.90) at a lag of −1 week, suggesting that PR became a highly reliable early indicator of morbidity trends in the aftermath of the COVID-19 pandemic (Figure 5).

### 3.3 Parameter selection

Under different δ values, the sensitivity ranges from 0.29 to 0.74, the specificity ranges from 0.87 to 0.98, the Positive Predictive Value (PPV) ranges from 0.75 to 0.89, the Negative Predictive Value (NPV) ranges from 0.71 to 0.86, and the Youden index (YI) ranges from 0.26 to 0.61. The parameter δ = 1.1 yields the best performance with the highest sensitivity, NPV, and YI (Table 3). The sensitivity, specificity, PPV, NPV, and Youden index are 0.74, 0.87, 0.76, 0.86, and 0.61, respectively. Therefore, δ = 1.1 was selected as the optimal parameter and applied in subsequent analyses.

**Table 3:** Model fitting effect under different parameter settings.

**Parameter δ**	**Sensitivity**	**Specificity**	**PPV**	**NPV**	**Youden index**
1.0	0.52	0.90	0.75	0.77	0.43
1.1	0.74	0.87	0.76	0.86	0.61
1.2	0.70	0.89	0.78	0.84	0.59
1.3	0.67	0.90	0.79	0.83	0.57
1.4	0.68	0.90	0.79	0.83	0.58
1.5	0.68	0.90	0.79	0.83	0.58
1.6	0.68	0.90	0.79	0.83	0.58
1.7	0.67	0.91	0.80	0.83	0.58
1.8	0.64	0.91	0.81	0.82	0.55
1.9	0.63	0.93	0.83	0.82	0.56
2.0	0.52	0.94	0.83	0.78	0.46
2.1	0.48	0.94	0.83	0.76	0.43
2.2	0.48	0.94	0.83	0.76	0.42
2.3	0.42	0.96	0.85	0.75	0.38
2.4	0.42	0.96	0.85	0.74	0.38
2.5	0.37	0.97	0.88	0.73	0.34
2.6	0.34	0.98	0.89	0.72	0.32
2.7	0.34	0.98	0.89	0.72	0.32
2.8	0.31	0.97	0.85	0.71	0.28
2.9	0.31	0.97	0.85	0.71	0.28
3	0.29	0.97	0.85	0.71	0.26

YI, Youden's Index; PPV, Positive Predictive Value; NPV, Negative Predictive Value.

### 3.4 Cross-validation of MEM

For the 2023–2024 season, the sensitivity, specificity, and YI were 0.93, 0.67, and 0.6, respectively. By the cross-validation process, the 2014–2015 season has the lowest sensitivity and YI. After excluding the data from the 2014–2015 epidemic season, the MEM models of each epidemic season performed well (Table 4). The data from 9 epidemic seasons (except 2014–2015) were finally included.

**Table 4:** Cross-validation results of influenza in Kunming before and after eliminating abnormal data from 2011–2012 to 2023–2024.

**Season**	**Incorporate all data**			**Excluding the 2014–2015 season**		
	**Sensitivity**	**Specificity**	**YI**	**Sensitivity**	**Specificity**	**YI**
2011–2012	0.73	0.90	0.63	0.73	0.90	0.63
2012–2013	0.87	0.87	0.74	0.87	0.87	0.74
2013–2014	0.64	0.86	0.50	0.64	0.86	0.50
2014–2015	0.20	0.99	0.19	–	–	–
2015–2016	0.59	0.85	0.44	0.59	0.85	0.44
2016–2017	0.86	0.63	0.49	0.94	0.58	0.52
2017–2018	0.86	0.78	0.63	0.88	0.76	0.64
2018–2019	0.98	0.53	0.51	0.99	0.51	0.50
2019–2020	0.89	0.97	0.86	0.89	0.97	0.86
2023–2024	0.93	0.67	0.60	0.96	0.65	0.61

YI, Youden's Index.

### 3.5 MEM for influenza seasons in Kunming

**Figure 6:**
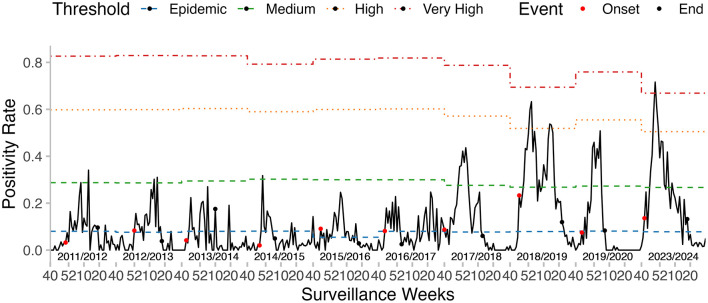
Influenza epidemic onset, duration, and thresholds of intensity determined by the MEM, in the seasons from 2011–2012 to 2023–2024.

The onset of the epidemic varied across seasons, ranging from week 40 to week 52, while the epidemic duration ranged from 11 to 29 weeks. The pre-epidemic threshold values were relatively stable, fluctuating between 5.49 and 8.10%. However, the thresholds for medium, high, and very high intensity exhibited noticeable variation across seasons. The majority of the seasons recorded medium or low intensity levels, except for 2018–2019, which was classified as high intensity, and 2023–2024, which reached a very high intensity level (Table 5, Figure 6).

**Table 5:** Influenza epidemic onset, duration, thresholds of PR, and peak intensity level determined by the MEM from 2011–2012 to 2023–2024 seasons.

**Seasons**	**Onset**	**Duration**	**Epidemic threshold (%)**				
			**Pre**	**Medium**	**High**	**Very high**	**Intensity**
2011–2012	50	22	8.03	28.74	59.79	82.65	Medium
2012–2013	52	19	7.61	28.65	59.87	82.92	Medium
2013–2014	43	20	8.02	29.45	60.32	82.80	Low
2014–2015	48	11	8.08	30.23	58.97	79.23	Medium
2015–2016	45	26	5.49	29.98	59.93	81.39	Low
2016–2017	44	12	8.01	29.97	60.16	81.87	Low
2017–2018	40	26	7.72	27.61	57.11	78.75	Medium
2018–2019	46	29	7.92	26.82	51.87	69.42	High
2019–2020	44	16	8.10	27.29	55.48	75.93	Medium
2023–2024	42	29	7.81	26.70	50.48	66.89	Very high

**Figure 7:**
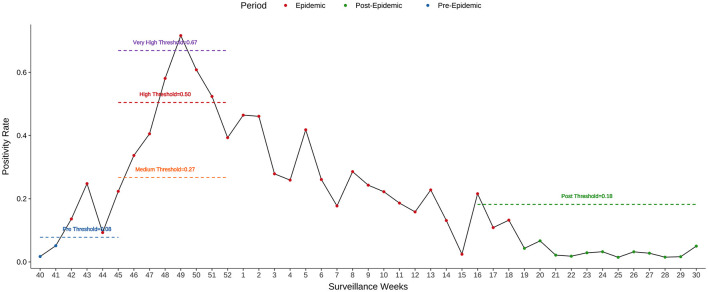
Weekly PR, MEM, and intensity for the 2023–2024 season in Kunming.

For the 2023–2024 influenza season, the epidemic period, as estimated by MEM, started in week 42 of 2023 and lasted for 29 weeks. The pre- and post-epidemic thresholds were 0.08 and 0.18, respectively (Figure 7). The timeliness of epidemic detection was zero weeks. There was no detection lag for the 2023–2024 season. The peak intensity was estimated at a very high level in this season.

### 3.6 Stratification analysis by influenza strain

In the analysis of influenza virus types from the 2011–2012 to the 2023–2024 seasons, a total of 3,870 positive samples were recorded. A(H1N1)pdm09 and A(H3N2) are the **two** dominant influenza strains in Kunming, accounting for 74.54% of positive samples. A(H1N1)pdm09 has the highest peak PR of 47.76% and was prevalent across 5 seasons, making it the most consistently dominant strain. In contrast, B/Yamagata-lineage and B/Victoria-lineage played a smaller role, with B/Yamagata-lineage being more prevalent, reaching a maximum peak of 38.04%. The MEM estimates indicate that influenza outbreaks typically start in late autumn or early winter (weeks 44–48) and last for about 16–18 weeks. The epidemic thresholds vary, with A(H3N2) having the highest threshold of 3% and B/Yamagata-lineage the lowest at 1.49% (Table 6).

**Table 6:** Epidemics by influenza virus strain in Kunming from 2011–2012 to 2023–2024 seasons.

**Influenza strain**	**Positive samples (*n*)**	**Percentage (%)**	**Maximum peak (%)**	**Moving epidemic model estimation**			
				***n* of seasons**	**Start week**	**Epidemic length (weeks)**	**Epidemic threshold (%)**
A(H1N1)pdm09	1,451	37.49	47.76	5	48	18	85
A(H3N2)	1,434	37.05	44.91	4	44	17	3
B/Yamagata-lineage	850	21.96	38.04	3	48	16	49
B/Victoria-lineage	135	59	27.27	1	44	–	–

## 4 Discussion

This study is the first to validate the Moving Epidemic Method (MEM) for influenza surveillance in a subtropical plateau setting. The MEM model exhibited robust performance, with high sensitivity (74%) and specificity (87%) for detecting epidemics, and no temporal lag in identifying the onset of the 2023–2024 influenza season. The epidemic threshold for the 2023–2024 season was determined at 8%, while subtype-specific thresholds varied markedly, 1.49–3%, underscoring distinct transmission dynamics among circulating strains. Notably, Kunming's threshold was substantially lower than those reported in other subtropical and tropical regions, such as Guangdong 11.99% and Wuhan 15.42% (24, 25), and even contrasted with the World Health Organization (WHO)-recommended baseline of 13.2% for tropical Cambodia (27). Although the exact impact is not yet clear (28–30), humidity, precipitation, and temperature are the primary climatic factors influencing tropical and subtropical regions. The spread of influenza in tropical and subtropical regions. Affected by the Kunming quasi-stationary front (31, 32), the climate in Kunming is sunny and dry during the winter and spring seasons. These discrepancies validate the adaptability of MEM in subtropical plateau environments but also emphasize the necessity of region-specific threshold calibration.

Spatiotemporal analysis revealed marked heterogeneity in influenza transmission across Kunming, with peripheral districts consistently exhibiting higher incidence rates than urban centers. Since 2018, overall influenza morbidity has sharply increased, culminating in a historical peak in 2023. This pattern suggests a potential exacerbation of pre-existing spatial inequalities in influenza burden, possibly reflecting disparities in healthcare access, population density, socioeconomic factors, or environmental conditions between urban core and peri-urban and rural regions. In parallel, cross-correlation analysis demonstrated that virological positivity rates (PR) consistently led reported incidence by ~1 week, particularly in the post-COVID-19 period. These findings highlight PR as a reliable and timely indicator of early warning indicator for influenza activity.

PR data can better reflect the real-world influenza epidemic situation compared to syndromic indicators, such as ILI (33, 34). Since the identification of influenza-like illness also includes other symptoms of viral infections, such as those caused by respiratory syncytial virus (RSV) and adenovirus (35–38). Therefore, virology-based data enhance specificity by minimizing confounding factors from non-influenza respiratory pathogens. Affected by the novel coronavirus, the PR has decreased in our surveillance data from 2020–2021 to 2022–2023. Our study, therefore, excluded these seasons at the beginning of data inclusion. Moreover, after the cross-validation process, the 2014–2015 season was removed due to its low sensitivity of 0.2. This low sensitivity might be attributed to poor surveillance quality, which led to false zero data occurring during the epidemic period. After the 2014–2015 season, we observed that the weekly PR values exhibited a more stable trend, and the occurrence of zero values between epidemic periods became less frequent. Additionally, the peak PR values showed an upward trend, increasing from 24.63 to 71.62% after the 2015–2016 season. In the 2023–2024 season, the influenza epidemic entered a low-intensity phase at the 42nd week. Subsequently, it rapidly transitioned to a high-intensity phase at the 48th week and reached its peak at the 49th week, with a PR of 71.62%. The high-intensity influenza epidemic was also detected in Beijing (39), and the number of infections has tripled in the 2023–2024 season.

The Moving Epidemic Method (MEM) shows superior performance in modeling single-peak epidemics compared to dual-peak patterns (20). In Kunming, influenza virology data predominantly exhibited a unimodal distribution across the majority of the seasons. When the absolute humidity (AH) is relatively low, the survival and transmission of influenza viruses will increase (40), which corresponds to the peak of influenza activity observed every winter. However, during the 2017–2018 and 2018–2019 seasons, a bimodal pattern emerged, characterized by a primary winter peak followed by a secondary spring peak. During these two seasons, the H1N1(pdm09) was the predominant circulating strain, accounting for over 40%, which was consistent with similar research conducted in Wuhan and Guangdong (24, 25). The situation has an obvious discrepancy between the two seasons. In the 2017–2018 season, the overall positivity rate (PR) trend was consistent with that of H1N1(pdm09). In contrast, during the 2018–2019 season, the bimodal pattern was caused by the trend of H1N1(pdm09) followed by that of B/Yamagata. The antigenic shifts in circulating influenza strains, which alter herd immunity dynamics and may prolong transmission windows (41). Therefore, stratified research on influenza is also of great significance. Through stratification analysis, the predominant strain was the A-type epidemic strain, and the B/Yamagata epidemic strain was also superimposed in some years. The H1N1(pdm09) has persisted for more than five seasons. In these seasons, it accounted for over 45%, and the epidemic duration in each of these seasons exceeded 20 weeks, determined by MEM. In contrast, when H3N2 became the predominant strain, the epidemic duration was 11–16 weeks, which was shorter than that of H1N1(pdm09). The B/Yamagata lineage mainly overlapped with the H3N2 strain during the 2023–2024 epidemic season. As for the B/Victoria lineage, it was only prevalent in the 2011–2012 season and not considered in MEM. The higher threshold for A(H3N2) (3%) compared to A(H1N1)pdm09 (2.85%) and B/Yamagata (1.49%) also reflects distinct transmission dynamics.

This study has several limitations. First, the generalizability of four sentinel sites might limit the findings to the urban city. The four sentinel surveillance sites were all located within Kunming's major district and remained unchanged from their establishment after the SARS outbreak in 2003 until 2024. This geographic concentration may limit the representativeness of our findings for the entire city, as the catchment areas of these sites do not fully encompass Kunming's diverse urban, peri-urban, and rural populations. Second, the limited representation of influenza B lineages precluded robust subtype-specific analyses. Third, the study did not include meteorological or population mobility data. Future research should integrate these factors to refine predictive models. Nevertheless, the absence of complementary data sources prevented us from exploring behavioral factors.

## 5 Conclusion

This study validates the MEM as a robust tool for influenza surveillance in subtropical plateau regions. By establishing localized thresholds and characterizing strain-specific dynamics, the findings support data-driven public health strategies aimed at mitigating the impact of seasonal influenza. Based on the MEM's performance, we recommend its integration into Kunming's routine influenza surveillance system. Subtype-specific thresholds should guide stratified responses, with increased vigilance during A(H3N2)-dominant seasons. Vaccination programs could be optimized by aligning administration with the typical onset of epidemics and prioritizing high-risk groups. Furthermore, the variability in B/Yamagata-lineage activity, coupled with its global decline after 2020, warrants continuous monitoring to assess potential resurgent risks. Future studies should focus on expanding data sources and exploring the applicability of MEM to other respiratory pathogens, thereby reinforcing global health preparedness in the face of evolving viral threats.

## Data Availability

The data analyzed in this study is subject to the following licenses/restrictions: the dataset is part of Kunming CDC Notifiable Communicable Disease Reporting System and the National Immunization Program System. It is not publicly available according to the legislations. Requests to access the datasets should be directed to YG, gao_yudong@163.com.
